# *Sonchus asper* (L.) Hill extracts: phytochemical characterization and exploitation of its biological activities by loading into nanoformulation

**DOI:** 10.3389/fpls.2024.1416539

**Published:** 2024-08-16

**Authors:** Valentina Parisi, Valentina Santoro, Immacolata Faraone, Nadia Benedetto, Antonio Vassallo, Nunziatina De Tommasi, Luigi Milella, Antonio Nesticò, Gabriella Maselli, Anna Maria Fadda, Carla Caddeo

**Affiliations:** ^1^ Department of Pharmacy, University of Salerno, Fisciano, Italy; ^2^ National Biodiversity Future Center (NBFC), Palermo, Italy; ^3^ Department of Science, University of Basilicata, Potenza, Italy; ^4^ Spinoff TNcKILLERS srl, Potenza, Italy; ^5^ Department of Civil Engineering, University of Salerno, Fisciano, Italy; ^6^ Department of Life and Environmental Sciences, University of Cagliari, Monserrato, Italy

**Keywords:** *Sonchus asper* (L.) Hill extract, eudragit-coated liposomes, antioxidant, hypoglycemic, GLP-1, economic evaluation

## Abstract

**Introduction:**

The current investigation presents a two-fold approach to rediscovering the potential of *Sonchus asper* as a wild edible plant, both in its raw extract form and as a nanoformulated product. Furthermore, the study aimed to promote the valorization of traditional dishes and contribute to biodiversity conservation and sustainable use of *S. asper*, thus enhancing economic profits.

**Methods:**

Liquid chromatography-mass spectrometry analyses were conducted to characterize the metabolite profile of the raw and cooked leaf extracts, and the extract from discarded leaves. The antioxidant activity, the hypoglycaemic effect and the incorporation into liposomes were evaluated.

**Results:**

38 compounds and 6 essential amino acids were identified. The incorporation into liposomes maximized the health-promoting properties for potential pharmaceutical or food applications.

**Discussion:**

The commercialization of *S. asper* could: (i) contribute to improving the well-being of rural and urban communities, being *S. asper* a wild edible plant available at low cost, environmentally friendly, resilient, and adaptable; (ii) generate landowner economic returns.

## 1 Introduction

Wild edible plants (WEPs) are a core element of eating habits worldwide (De Cortes Sánchez-Mata and Tardío, 2016). The Food and Agriculture Organization (FAO) estimates that, in the European Union, around 20% of the population (100 million people) consumes wild foods, while 14% (corresponding to more than 65 million people) harvest wild plants at least occasionally (Bacchetta et al., 2016). WEPs are considered functional foods, in fact, they can promote health apart from possessing their nutritional value (Bermejo and León, 1994). Moreover, as indigenous species thrive in their original habitat, they contribute to the regions’ identity, traditions, and cultural history.

Engaging in the production and harvest of WEPs offers numerous socio-cultural, healthy and economic benefits to local farmers and communities. According to Bacchetta et al. (2016), WEPs contribute to diversifying agronomic productivity and improving the different crops’ resilience (Bacchetta et al., 2016). While the precise impact of regular WEP consumption in preventing diet-related diseases remains uncertain, wild plants generally exhibit higher levels of micronutrients, specialized active metabolites, and vitamins C and A, as well as the greatest amount of minerals, protein, and fiber concentrations compared to cultivated plants (Heinrich et al., 2006; Amirul Alam et al., 2014). WEPs may also serve as proto-dietetic supplements with potential chemo- and cardio-preventive properties (Visioli et al., 2004; Bondonno et al., 2015). Besides their health-related properties, WEPs exhibit high genetic variability, which confers resilience to dryness and climate change, and high tolerance to pests and diseases (Dempewolf et al., 2008). Despite their potential to improve nutrition, agrobiodiversity, food security, and welfare, there is still a significant lack of information and knowledge on the properties of WEPs. Consequently, their significant contributions to the human diet have not yet been sufficiently recognized.

In addition, WEPs are increasingly threatened by urbanisation and the globalisation of agriculture (Panfili et al., 2020) or, as in the case of the inland areas of Mediterranean regions particularly Southern Italy, by depopulation and the consequent spread of fallow land. Therefore, actions and strategies useful for the economic valorization of WEPs are urgently needed to preserve native wild species.

In this context, *Sonchus asper* (L.) Hill, a species belonging to the Asteraceae family, is a potential wild edible species to valorize. *S. asper* is an erect, robust, spiny annual or biennial herb up to 1.8 m high and characterized by leaf rosette and yellow flowers. Its flowering period is from October to December or January. It is a weed of cultivated fields but can also be found in dunes, valleys, seasonal wetlands, along lakeshores, and on mud, at 750–2550 m altitude (Pignatti et al., 2017). *S. asper* is among the plants most used in traditional Mediterranean cuisine and is typically eaten as a soup or side dish by people in southern Italy’s inland areas (De Cortes Sánchez-Mata and Tardío, 2016). In Benevento province, the plant is commonly named “*Cardillo*” and is widely consumed traditionally in soups such as the traditional “*zuppa delle streghe*”.

Several scientific studies have highlighted the beneficial properties of *S. asper* attributable to the polyphenols, terpenes, and carotenoids in fresh leaves and extracts (Khan et al., 2014; Panfili et al., 2020; Altin et al., 2021; Fratianni et al., 2021). For this reason, *S. asper* preparations are widely used for treating numerous human illnesses, such as cough, gastrointestinal infections, diabetes, wounds and burns, and inflammatory diseases (Xia et al., 2011; Jain et al., 2015; Khan, 2017).

In this study, the extraction of high added-value compounds (phenols, flavonoids, fatty acids, amino-acids) from *S. asper* edible leaves, both raw and cooked, was carried out by green extractions, such as ultrasound-assisted extraction (U) and microwave-assisted extraction (MW) using hydroalcoholic solvents. As far as we are aware, this is the first time that the quali-quantitative characterization of metabolites contained in *S. asper* leaves has been conducted. The *in vitro* antioxidant activity of *S. asper* extracts was also performed. In addition, given the nutritional potential of these extracts, the external leaves from *S. asper*, which are usually discarded after harvest, were also studied. They were subjected to ultrasound-assisted extraction, and the antioxidant and hypoglycemic activities were investigated by *in vitro* and cellular assays. For a potential application in healthy and nutritional fields, a new delivery system of the extract from discarded non-edible leaves was created by developing eudragit-coated liposomes in order to provide protection during transit in the gastrointestinal tract, improving the bioavailability and efficacy of the extract’s bioactive compounds.

Furthermore, we show that the promotion of actions and strategies to preserve native wild species generates economic returns to both landowners and the community. Particularly, this investigation analyzed a cooperation scheme between an agricultural consortium responsible for the harvesting and marketing of *S. asper*, and the owners of abandoned land that allows the harvesting of WEPs. This economic scheme not only allows for the productive utilization of marginal agricultural land in marginal areas of Mediterranean region (and other areas with similar socio-economic structures), but also leads to positive spin-offs in terms of employment and cultural enhancement of local traditions.

## 2 Materials and methods

### 2.1 Chemicals and reagents

6-Hydroxy-2,5,7,8-tetramethylchroman-2-carboxylic acid (Trolox), Folin-Ciocalteu, 2,2-diphenyl-1-picrylhydrazyl (DPPH), 2,4,6-tris(2-pyridyl)-*s*-triazine, *α*-amylase enzyme from porcine pancreas, starch, iodine (I_2_), potassium iodide (KI), 4-*p*-nitrophenyl-*α*-d-glucopyranoside, acarbose, α-glucosidase enzyme from *Saccharomyces cerevisiae*, glutamine, potassium phosphate monobasic, fetal bovine serum (FBS), sodium carbonate, 2-deoxy-2-[(7-nitro-2,1,3-benzoxadiazol-4-yl)amino]-d–glucose (2-NBDG), Dulbecco’s modified Eagle’s medium (DMEM), streptomycin, penicillin, and 3-(4,5-dimethylthiazol-2-yl)-2,5-diphenyltetrazolium bromide (MTT) were purchased from Merck (Milan, Italy). Glucagone like peptide-1 (GLP-1) Elisa kit was purchased from Invitrogen. Water, methanol, and acetonitrile for liquid chromatography mass-spectrometry (LC-MS) were acquired from Romil Ltd Pure Chemistry (Cambridge, United Kingdom). For quali-quantitative analysis the following standards were used: luteolin 7-*O*-glucoside and linoleic acid were purchased from Cayman Chemical (Michigan, USA); rutin, chlorogenic acid, apigenin 7-*O*-glucoside, and aesculetin, L-threonine, L-phenilalanine, L-tryptophan, L-leucine. L-lysine and L-isoleucine analytical standards (≥98% by HPLC) were obtained from Sigma-Aldrich (Milan, Italy); roseoside as reference standard was obtained by HPLC from plant material. Phospholipon90G (>94% phosphatidylcholine) was from Lipoid GmbH (Ludwigshafen, Germany); Eudragit^®^ L100 (1:1 methacrylic acid-methyl methacrylate copolymer) was from Evonik Industries AG (Essen, Germany); solvents for extraction, phosphate buffered saline and stearylamine were purchased from Sigma-Aldrich/Merck (Milan, Italy).

### 2.2 Collection of plant material and extraction procedures

*S. asper* (Asteraceae) was collected in Contrada San Chirico (41°09’12.2”N 14°46’41.1”E), Benevento (Campania, Italy) during the spring of 2021, and a good specimen (N-A. 9595) was deposited at the University of Pisa Herbarium (Italy). The plant was harvested during the preflowering stage, according to the traditional use. Fresh leaves and young stems were chopped in a commercial blender; an aliquot of these edible parts was cooked in boiling water for 2 min, according to the cooking method used in the traditional recipe, and dried with paper towels. MW was performed on either raw or cooked materials (20 g) using a modified microwave cavity apparatus (Whirlpool MWF 426 SL, 800 W; cavity size: L 31.7 cm; H 21 cm; W 31.5 cm). The microwave power was controlled using a specially designed power supplier (5 kV, 1,000 W maximum power) with continuous modulation of current intensity. A rotating paddle ensured the homogenization of the microwave field, avoiding the need for a rotating plate. Optical fibers Optocon^®^ (-200°C/+300°C) were used to monitor the sample temperature. The extraction process lasted 5 min. U of both the cooked and the raw material (20 g each) was carried out using a 320 W Ultrasonic bath (Branson 2510E-MTH, Bran-sonic^®^, Milan, Italy). Extractions were carried out using the mixture of EtOH:H_2_O 7:3 and plant material:solvent ratio of 1:10 (*w/v*) for 15 min.

After drying and grinding, *S. asper* discarded leaves (SAD) (non-edible external hard leaves) were extracted with EtOH:H_2_O 7:3 mixture using an ultrasound bath (320 W Ultrasonic bath) and plant material to solvent ratio of 1:10 (*w/v*), for 15 min. Subsequently, the extract underwent exhaustive maceration to enhance its richness in active metabolites.

After filtration, extracts were dried under vacuum, frozen, and freeze-dried to eliminate the excess water. They were then kept at 4°C for subsequent analysis.

### 2.3 LC-HRMS analysis: quali-quantitative analyses of *S. asper* specialized metabolites

All dried extracts were solubiized in MeOH:H_2_O (4:1), centrifuged for 10 min at 13000 rpm and injected in LC-MS apparatus for qualitative determinations. Qualitative profiles of the extracts obtained from raw and cooked plant were obtained by Q Exactive™ Hybrid Quadrupole-Orbitrap™ Mass Spectrometer Q-trap (Thermo Fisher Scientific, Milan, Italy) coupled with an UltiMate 3000 UHPLC system (Thermo Fisher Scientific). The HRMS data were taken in negative ion mode and the ESI-MS/MS experiments were done using 35.0% normalized collision energy. The capillary temperature was fixed at 320°C, auxiliary gas and flow rate of sheath gas were regulated at 15 and 35.0 arbitrary units, respectively. A C18 column (Luna C18, Phenomenex, 150 × 2.0 mm, 3 μm) and a binary mobile phase [eluent A (ultrapure water–0.1% *v/v* formic acid) and eluent B (ultrapure acetonitrile)] were used. The separation conditions were set as follows: an isocratic step at 5% of B for 5 min, followed by a first gradient from 5% to 50% of B in 45 min and a faster gradient from 50% to 100% of B in 10 min. Flow rate was 0.2 mL/min and the injection volume was 10 μL. The same analytical set up was used to quantify, in the extracts obtained from the edible parts, the main identified compounds belonging to different chemical classes. Different calibration curves, in a concentration range from 1 ng/mL to 1 μg/mL, were set up, using the following standards: chlorogenic acid, apigenin 7-*O*-glucoside, luteolin 7-*O*-glucoside, luteolin, rutin, aesculetin, roseoside and alpha linolenic acid to quantify phenolic acids, coumarines, roseoside derivatives and fatty acids. The stock solutions (1 mg/mL) of each pure compounds were prepared and at least seven different concentrations obtained by serial dilutions were injected. Analyses were performed in triplicate and the results are reported as means ± standard deviations (Parisi et al., 2022).

### 2.4 LC-HRMS analysis: quali-quantitative analyses of *S. asper* aminoacids

The quali-quantitative determination of essential amino acids was carried out using a Q Exactive™ Hybrid Quadrupole-Orbitrap™ Mass Spectrometer (Thermo Fischer Scientific Inc., Darmstadt, Germany) operating in positive ion mode coupled with the Thermo Scientific UltiMate 3000 UHPLC system. A Luna^®^ C18 150 × 2 mm, 3 µm (100 Å) column (Phenomenex^®^, Castel Maggiore, Bologna, Italy) was employed. The mobile phase consisted of H_2_O acidified by 0.1% formic acid *v/v* (solvent A) and CH_3_CN acidified by 0.1% formic acid *v/v* (solvent B) with the following linear gradient as elution method: a first isocratic step of 2% of solvent B of 5 min followed by a faster gradient from 2% to 20% of solvent B in 8 min. The flow rate was 0.2 mL/min, and the column oven was set to 25°C. The calibration curves for lysine, tryptophan, phenylalanine, threonine, isoleucine, leucine, methionine, and valine were built in a concentration interval from 20 ng/mL to 2 μg/mL. The instrument response linearity in the tested concentration interval was verified for each compound. Experiments were performed in technical triplicates.

### 2.5 Production and characterization of vesicles

For the production of liposomes, a simple procedure that involves the dispersion of the components in aqueous medium and the sonication was applied. Phospolipon90G, stearylamine, and the *S. asper* extract obtained from discarded non-edible leaves (SAD) were dispersed in Phosphate Buffered Saline and sonicated (5 sec on and 2 sec off, 15 cycles + 3 sec on and 2 sec off, 12 cycles) with a disintegrator (Soniprep 150 plus, MSE Crowley, London, UK). Thereafter, the liposome dispersion was added dropwise, under gentle stirring, to an equal volume of 0.1% *w/v* eudragit solution to produce eudragit-coated liposomes (Caddeo et al., 2019). Empty uncoated liposomes and empty eudragit-coated liposomes were produced according to the same procedure, but without using SAD (**Table 1**).

**Table 1:** Composition and main features of the liposome formulations: mean diameter (MD), polydispersity index (P.I.), zeta potential (ZP), and entrapment efficiency (E).

Formulation	P90G	SA	*SAD*	PBS	Eu in PBS (0.1% *w/v*)	pH	time	MD nm ± SD	P.I.	ZP mV ± SD	E % ± SD
**Empty liposomes**	120 mg	6 mg		1 mL				80 ± 5.6	0.25 ± 0.02	+8 ± 0.8	–
**SAD liposomes**	120 mg	6 mg	4 mg	1mL				81 ± 4.2	0.24 ± 0.02	+7 ± 0.6	–
**Empty eu-liposomes**	120 mg	6 mg		1mL	1 mL			^**^103 ± 13.9	^**^0.36 ± 0.06	^**^+6 ± 1.4	–
**SAD eu-liposomes**	120 mg	6 mg	4 mg	1mL	1 mL			^**^108 ± 15.2	^**^0.39 ± 0.05	^**^+6 ± 0.9	Luteolin-glucuronide 93 ± 3.2 Apigenin-glucuronide 94 ± 2.3
Incubation with gastrointestinal media at 37°C.											
**SAD liposomes**						1.2	**t_0_**	91 ± 7.5	0.29 ± 0.08	+10 ± 0.8	
							**t_2h_**	97 ± 2.9	0.35 ± 0.01	+11 ± 1.0	
						7.0	**t_0_**	105 ± 9.0	0.31 ± 0.03	+4 ± 1.4	
							**t_6h_**	103 ± 3.6	0.32 ± 0.05	+4 ± 0.3	
**SAD eu-liposomes**						1.2	**t_0_**	94 ± 2.6	0.31 ± 0.09	+11 ± 0.7	
							**t_2h_**	94 ± 1.2	0.29 ± 0.06	+10 ± 0.7	
						7.0	**t_0_**	90 ± 0.7	0.31 ± 0.05	+4 ± 1.0	
							**t_6h_**	97 ± 4.3	0.34 ± 0.07	+2 ± 0.1	

Mean values ± SDs are reported (n = 10). ^**^ values statistically different (p < 0.01) from uncoated liposomes.

MD, P.I. and ZP of *S. asper* liposomes and eudragit-coated liposomes are reported upon dilution (t_0_) with gastrointestinal media (pH 1.2 or 7.0) and incubation at 37°C for 2 h (t_2h_) or 6 h (t_6h_). Mean values ± SDs are reported (n = 4).

P90G, phospholipid; SA, stearylamine; SAD, *S. asper* extract from discarded non-edible leaves; PBS, phosphate buffered saline; Eu, Eudragit^®^ L100.

For the characterization of the vesicles, the determination of the average diameter, the polydispersity index, and the zeta potential of the vesicles, the dynamic and electrophoretic light scattering techniques were applied by means of a Zetasizer nano-ZS (Malvern Panalytical, Worcestershire, UK).

For the purification from the non-incorporated extract compounds, the SAD eu-liposomes (2 mL) were dialyzed against PBS (1 L) for 2 h using Spectra/Por^®^ membranes (12–14 kDa MW cut-off; Spectrum Laboratories Inc., Breda, The Netherlands). Both non-dialyzed and dialyzed vesicles were disrupted by diluting (1:100 *v/v*) with methanol and analyzed by LC-MS (see Section 2.3) to quantify luteolin-glucuronide and apigenin-glucuronide and calculate the entrapment efficiency (E).

### 2.6 Behaviour of the vesicles in gastrointestinal environment

The behaviour of SAD eudragit-coated liposomes in the mimicked gastrointestinal environment was studied. The average diameter, the polydispersity, and the zeta potential were measured after dilution (1:100 *v:v*) of the SAD eudragit-coated liposomes with an acidic medium (0.1 M HCl, pH 1.2) mimicking the gastric fluid or with a neutral medium (pH 7.0) mimicking the intestinal fluid, and after 2 or 6 h of incubation, respectively, at 37°C. Sodium chloride (0.3 M) was added to both media for ionic strength regulation. SAD uncoated liposomes were also tested to evaluate the protective effect of the eudragit coating.

### 2.7 Total phenolic content of *S. asper* edible and discarded leaves

The content of phenolic compounds (TPC) was evaluated by the Folin-Ciocalteu assay (Da Pozzo et al., 2018; Labanca et al., 2020). For the experiments, 75 µL of the *S. asper* hydroalcoholic extracts (i.e., raw MW, cooked MW, raw U, and cooked U) and SAD, in solution or in empty eudragit-coated liposomes, and eudragit-coated liposomes, 500 µL of Folin-Ciocalteu reagent and 500 µL of 10% *w/v* aqueous Na_2_CO_3_ were added into a microcentrifuge tube and water was made to achieve the final volume of 1500 μL. The absorbance was read at 723 nm employing a UV-visible spectrophotometer (SPECTROstar^Nano^ BMG Labtech, Ortenberg, Germany). The total content of phenols was indicated as mg of gallic acid equivalents (GAE) per g of dried extract (DW) or mL of solution (for liposome formulation) by using a calibration curve.

### 2.8 *In vitro* antioxidant activity of *S. asper* edible and discarded leaves

The antiradical activity and the reducing power of SAD, in solution or in eudragit-coated liposomes (2 mg/mL), and the *S. asper* hydroalcoholic extracts (i.e., raw MW, cooked MW, raw U, and cooked U) were performed by different *in vitro* colorimetric assays. Empty eudragit-coated liposomes were also tested to determine the possible activity of the vehicle. The antiradical scavenging activity was performed by the 2,2-diphenyl-1- picrylhydrazyl (DPPH) radical test (Labanca et al., 2020): a radical methanolic solution (100 µM; 200 µL) was added to each sample (50 µL) and incubated at room temperature in the dark (30 min). The absorbance was measured at 515 nm and results were expressed as mg Trolox equivalents per g of dried extract or mL of solution by using a calibration curve of Trolox standard. The reducing power was evaluated by the Ferric Reducing Antioxidant Power (FRAP) assay (Faraone et al., 2020). Each sample (25 µL) of was mixed to a TPTZ–ferric solution (225 µL). After an incubation at 37°C for 40 min in the absence of light, the absorbance was measured at 593 nm. The obtained data were expressed as mg Trolox equivalents per g of dried extract or mL of solution by interpolation of a Trolox standard curve.

The Relative Antioxidant Capacity Index (RACI) was employed as a statistical method to combine the outcomes obtained from the antioxidant assays performed *in vitro.* RACI is an arbitrary index that compares the average and the standard deviation of antioxidant methods’ raw data. By determining the standard score, which indicates the deviation of the raw data from the mean in terms of standard deviation units, it is possible to establish whether the raw data is lower or higher than the mean. In cases where the raw data is smaller than the mean, the standard score will be negative, and vice versa. A histogram was used to present the RACI results (Faraone et al., 2020). This histogram visually represents the final RACI data, providing a comparative overview of the antioxidant capacity across different samples or treatments.

### 2.9 Inhibition of the carbohydrate-hydrolyzing enzymes

The inhibition of *α*-amylase enzyme was carried out by using the KI/I_2_ method (Uddin et al., 2022) while the inhibition of *α*-glucosidase was performed by the substrate *p*NPG conversion into *α*-d-glucose and *p*-nitrophenol (Faraone et al., 2021). Assays were performed in triplicate.

### 2.10 Cell culture condition

STC-1 cells, a cell line derived from intestinal enteroendocrine cells (CRL-3254™), were acquired from the American Type Culture Collection through LGC Standards (Wesel, Germany). The cells were cultured in Dulbecco’s modified Eagle’s medium (DMEM) and supplemented with 10% fetal bovine serum (FBS), streptomycin (100 μg/mL), penicillin (100 units/mL) and 2 mM glutamine, and stored with 5% CO_2_ at 37°C in a humidified atmosphere. The SAD was dissolved in DMSO and diluted to the tested dilution with fresh medium. In all the experiments, as control, DMSO-treated cells (0.4% *v/v*) were used. The eudragit-coated liposomes were diluted with fresh medium to reach the required concentrations of SAD. For the experiments, cells were grown until a confluence of 70–80%.

### 2.11 Cell viability assay

The effect of *S. asper* extract, in solution or in eudragit-coated liposomes, and empty eudragit-coated liposomes on cell viability was evaluated by the MTT colorimetric assay (Sinisgalli et al., 2020). The cells were seeded into 96-well plate (2.0 ×10^4^ cells/well) for 48 h and then treated for 2 h with different SAD concentrations (1-200 µg/mL). The medium was then replaced by an MTT solution in DMEM (0.75 mg/mL) for 4 h. The formazan crystals generated by the viable cells were solubilized with a solubilization mixture (1:1 DMSO:isopropanol). The absorbance was spectrophotometrically determined at 560 nm employing a UV–Vis spectrophotometer.

### 2.12 Intestinal glucose uptake

To assess the impact of *S. asper* extract and eudragit-coated liposomes on glucose uptake, 1.3 × 10^4^ cells were seeded into a 96-well black plate, with clear bottom, for 24 h. The cells were then maintained in serum-free medium for 24 h before adding the test samples. The culture medium was then discarded and the cells were washed 2 ways with glucose-free, serum-free medium and test samples (1-10 µg/mL) were added to the cells. After a 2-hour incubation period, cells were washed with PBS two times and 100 nM 2-NBDG was added for 30 min. Subsequently, cells were washed with ice-cold PBS to prevent 2-NBDG efflux for two times and fluorescence was measured by using the microplate reader GLOMAX Multidetection System (Promega, Madison, WI, USA) (λex = 460 to 490 nm, λem =530 to 550 nm). The 2-NBDG uptake by cells was expressed as % of control cells (Yamamoto et al., 2015).

### 2.13 GLP-1 secretion assay

For experiments on GLP-1 secretion, STC-1 cells were seeded at a density of 2.0 × 10^6^ cells/well into 12-well culture plates, and once achieved the 70–90% of confluence, they were incubated with different SAD concentrations, in solution or in eudragit-coated liposomes (1-10 µg/mL). After an incubation of 2-hour, the culture medium was collected and subjected to centrifugation to eliminate cellular debris. The supernatants obtained were then kept at -80°C until further analysis. To assess GLP-1 secretion, a GLP-1 ELISA kit (Invitrogen BMS2194) was employed following the manufacturer’s instructions. This method enabled the quantification of GLP-1 levels in the collected supernatants, providing insights into the impact of the SAD treatments on GLP-1 secretion by the STC-1 cells.

### 2.14 Analysis of the economic viability of a consortium for the valorisation and marketing of *S. asper*

Considering the potential benefits of *S. asper* on the human health and environment, it is becoming increasingly urgent to develop a participatory and holistic approach to the use of WEPs. National and international bodies should jointly promote agricultural policies that encourage the use of WEPs.

Among the possible actions, this study was focused on one based on cooperation between agricultural consortia and landowners. To assess the financial feasibility of this strategy, the annual monetary revenues due to the sale of the WEP by the consortium’s operators at local markets are compared with the monetary disbursements corresponding to the value of the resources, goods and services annually employed to market the *S. asper*. It grows spontaneously and does not require irrigation. Therefore, the costs to be considered concern: (a) the cost of labour for harvesting; (b) the cost of transport to the selling market; (c) the fee to be paid by the farmers’ consortium to the landowners allowing the consortium to harvest WEPs.

### 2.15 Statistical analysis

Data are expressed as means ± SDs and analysis was done using the one-way ANOVA followed by Tukey’s *post-hoc* test (GraphPad Prism software, version 8.1).

## 3 Results

### 3.1 *S. asper* extracts qualitative analysis

**Figure 1:**
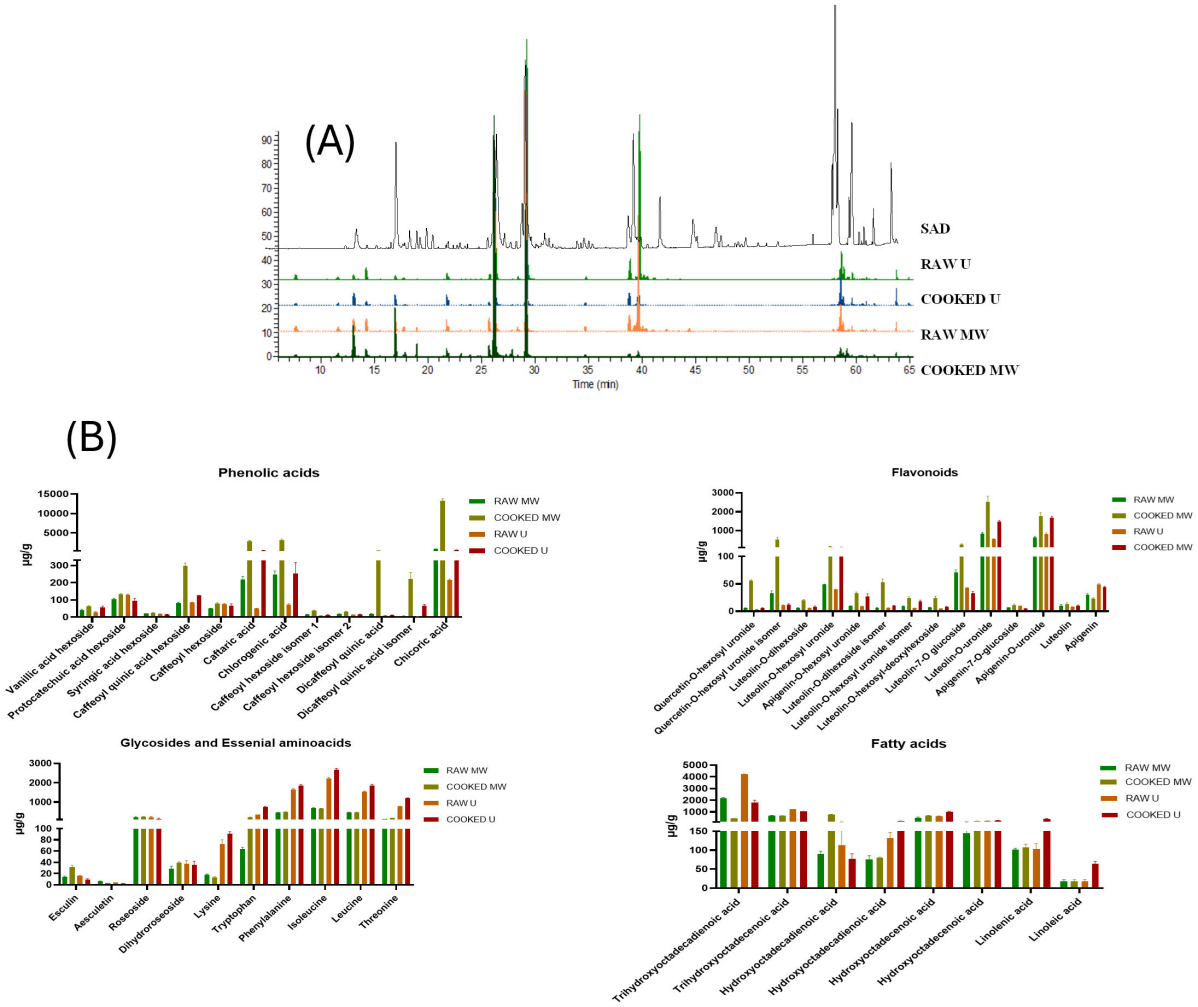
LC-HRESIMS profile of *S. asper* extracts from raw and cooked edible parts obtained by U and MW extraction **(A)** and LC-HRESIMS chromatograph of *S. asper* SAD acquired in negative ion mode **(B)** Quantitative analysis of *S. asper* extracts expressed as µg/g of dried extract.

The hydroalcoholic extracts derived from raw and cooked *S. asper* edible parts, and extracts obtained from SAD, were submitted to LC-HRMS analysis to investigate their phytochemical profiles. The specialized metabolite composition was very similar for all the extracts, as shown in **Figure 1A**.

Through the utilization of mass accuracy value, tandem mass experiment results, and information from existing literature, a total of 38 constituents were tentatively identified. The high-resolution mass values exhibited deviations of no more than 5 ppm in comparison to the calculated exact mass for each molecule. Some compounds were identified, referring to the laboratory chemistry library standards. Additionally, previously isolated and characterized compounds from authenticated plant materials using NMR and MS data were used for comparison. A comprehensive list of all the identified compounds is reported in **Table 2**. As reported, *S. asper* extracts are a complex phytochemical matrix with compounds belonging to different chemical classes. The first region of the chromatograms (**Figure 1A**) was characterized by the presence of phenolic acids (1-12, **Table 2**). According to the observed fragmentation pattern, compounds 4-12 were identified as caffeic acid derivatives, as stated by their fragment ion at *m/z* 179, which represents a deprotonated caffeic acid. In particular, compounds 5, 8, and 9 (t_R_ = 12.9, 16.9, and 17.0 min, respectively) were caffeoyl acids glycosylated with a hexoside, as evidenced by the presence of a product ion [M−H−162]^−^ at *m/z* 179 generated by the parent ion [M−H]^−^ at *m/z* 341.0867 fragmentation. Compounds 7 and 10 (*m/z* 353.0880 [M−H]^-^), were quinic acid derivatives, as can be deduced from the the fragment ion at *m/z* 191 [M–H-162]^-^. Furthermore, compound 7 identity was confirmed by the respective authentic standard injection. Compound 4 (t_R_ = 11.9 min) was a caffeoyl quinic acid derivative glycosylated with a hexoside, as deduced by the product ion at *m/z* 191 generated by parent ion 515.1407 [M−H]^-^fragmentation. The presence of dicaffeoylquinic acid (11) was highlighted by the ESI mass spectrum displaying a deprotonated molecule [M−H]^−^ at *m/z* 515.1193 and fragment ions at *m/z* 353,179 and 191, characteristic of a dicaffeoylquinic acid. At the retention time of 27.8 min, chicoric acid (12) was identified (*m/z* 473.072 [M−H]^-^). The fragmentation pattern of the [M−H]^−^ ion showed diagnostic product ions at *m/z* 179 (elemental composition C_9_H_8_O_4_), 149 (elemental composition C_4_H_5_O_6_), and 133 (elemental composition C_8_H_7_O_2_), which are consistent with those reported in the literature (Diao et al., 2018).

**Table 2:** Qualitative analysis of tentatively identified compounds detected in hydroalcoholic *S. asper* extracts and discarded leaves extract (SAD).

Peaks	t_R_	Compounds	[M-H]^-^	MS^2^	MSI status^a^
Phenolic acids					
1	7.6	Vanillic acid hexoside	329.0882	167, 152	2
2	7.7	Protocatechuic acid hexoside	315.0722	153, 109	2
3	11.1	Syringic acid hexoside	359.0986	197, 153	2
4	11.9	Caffeoyl quinic acid hexoside	515.1407	191, 179, 161	2
5	12.8	Caffeoyl hexoside	341.0867	179, 135	2
6	12.9	Caftaric acid	311.0410	179, 149, 135	2
7	16.6	Chlorogenic acid	353.0880	191, 179	1
8	16.9	Caffeoyl hexoside isomer 1	341.0867	179, 135	2
9	17.0	Caffeoyl hexoside isomer 2	341.0867	179, 135	2
10	19.4	Caffeoyl quinic acid isomer	353.0880	191, 179	2
11	27.2	Dicaffeoyl quinic acid	515.1193	353, 179, 191	2
12	27.8	Chicoric acid	473.0718	179, 149, 133	2
Flavonoids					
13	17.9	Quercetin-*O-* hexosyl uronide	639.1210	301, 463	2
14	19.3	Quercetin-*O-* hexosyl uronide isomer	639.1210	301, 463	2
15	21.3	Luteolin-*O-* dihexoside	609.1459	285	2
16	21.5	Luteolin-*O-* hexosyl uronide	623.1249	285	2
17	22.1	Apigenin-*O-* hexosyl uronide	607.1307	269, 431	2
18	22.8	Luteolin dihexoside isomer	609.1459	285	2
19	24.1	Luteolin-*O-* hexosyl uronide isomer	623.1249	285	2
20	24.9	Luteolin-*O-* hexosyl deoxyhexoside	593.1512	285	2
21	25.7	Luteolin-7-*O*-glucoside	447.0935	285	1
22	26.3	Luteolin-*O-*uronide	461.0970	285	2
23	28.2	Apigenin-7-*O*-glucoside	431.0724	269	1
24	29.2	Apigenin-*O-*uronide	445.0771	269	2
25	34.4	Luteolin	285.0407	193, 149	1
26	38.7	Apigenin	269.0454	179, 191. 353	2
Coumarins and glycoside					
27	13.7	Esculin	339.0721	177, 133, 105	1
28	17.1	Aesculetin	177.0185	133, 105	2
29	19.8	Roseoside	385.1866	205, 135	1
30	25.5	Dihydroroseoside	387.0756	207	2
Fatty acids					
31	35.8	Trihydroxyoctadecadienoic acid	327.2180	229, 211	2
32	42.1	Trihydroxyoctadecenoic acid	329.2116	229, 211	2
33	58.5	Hydroxy C18:3	291.2000	185, 121	2
34	58.7	Hydroxy C18:3	291.2000	185, 121	2
35	59.0	Hydroxy C18:2	293.2115	275, 195	2
36	59.3	Hydroxy C18:2	293.2115	275, 195	2
37	63.6	Linolenic acid	277.2160	259, 231, 181	1
38	64.8	Linoleic acid	279.2322	261, 209, 187	2
Peaks	t_R_	Compounds	[M+H]^+^	MS^2^	MSI status^a^
Essential aminoacids^b^					
39	1.35	Lysine	147.1128	130, 79	1
40	1.66	Threonine	120.0655	120, 79	1
41	3.58	Isoleucine	132.1019	86, 79	1
42	3.94	Leucine	132.1019	86, 79	1
44	8.35	Phenylalanine	166.0862	120, 79	1
45	12.7	Tryptophan	205.0971	188, 79	1

a MSI level of identification according to (Summer et al., 2007).

b Amino acids were confirmed by methods reported in experimental section.

The same criteria were used to identify the flavonoid derivatives (13-26). Derivatives of luteolin, quercetin, and apigenin were found in all the extracts. Among them, compounds 15, 16, 18-22, displayed in the MS/MS spectra the identical diagnostic product ion at *m/z* value of 285 [M−H]^-^, matching with luteolin (25). According to the MS/MS fragmentation and reference standards, these compounds were recognised as luteolin 7-*O*-glucosides, when the loss of a single hexose (162 Da) was observed in the MS/MS spectrum, or luteolin-dihexosides, when a loss of 304 Da corresponding to two *O*-linked hexose units was revealed. Moreover, luteolin-uronide (22) and luteolin-hexoside-uronide (16) were also identified, based on the base ion peak at *m/z* 285 [M−H]^-^ observed in the MS fragmentation of 22, corresponding to the loss of one uronic acid unit, and the subsequent loss of one hexose (162 Da) and one uronic acid (176 Da) observed in the spectrum of 16. Based on MS/MS fragmentation pattern, assigning the exact position and identity of the sugar units was not possible. Compounds 13 and 14 were identified as flavonol glycosides, due to the presence in the MS^2^ spectra of the ion at *m/z* value of 301 [M−H]^-^, corresponding to quercetin aglycon. Compounds 17 (t_R_ = 22.1 min), 23 (t_R_ = 28.2 min), and 24 (t_R_ = 29.2 min) were three apigenin glycosides, as demonstrated by the presence in the MS^2^ spectra of an ion at *m/z* 269 [M−H]^-^. This product ion, corresponding to deprotonated apigenin, was generated by the loss of one hexose for 23, of one uronic acid for 24, and by the subsequent loss of one hexose and one uronic unit for 17. Compound 23 identity was confirmed by injection of an authentic standard, while compounds 17 and 24 were assigned to apigenin derivatives, but it was not possible to assign the sugar moiety position. Moreover, two coumarins, esculin (27) and aesculetin (28), and C13-norisoprenoid derivatives, roseoside (29) and dihydroroseoside (30) were also identified. Compounds 27 and 29 were confirmed by injection of an authentic standard. In the MS/MS analysis of 28, a subsequent loss of CO, corresponding to the [M-H-CO]^-^ *m/z* 149, and the [M-H-CO_2_]^-^ *m/z* 133 typical of aesculetin fragmentation pattern (Li et al., 2012) were found. Compound 30 showed a molecular ion peak [M-H]^-^ at *m/z* 387.0756. The presence of one hexose unit was suggested by a fragment ion at *m/z* 207 [M–H-18-162]^-^, while the fragment at *m/z* 137 [M-H-162-18 -70]^-^ was due to the subsequent loss of C_4_H_6_O side chain. The fragmentation of 30 produced peaks coinciding with that of 29, with a difference of 2 mass units (**Table 2**). Based on data comparison, compound 30 was identified as 7,8-dehydro-6-hydroxy-3-oxo-*α*-ionol hexoside (dihydroroseoside) (Mina et al., 2016). Several fatty acids, such as linolenic (37) and linoleic acids (38), and their mono and tri-hydroxylated derivatives (31-36) were also identified in the extracts. Examination of the ESI-MS/MS data of two 18-carbon fatty acids, linoleic (18:2) and *a*-linolenic (18:3) acids, showed the top part of the spectrum to be dominated by the loss of H_2_0 (*m/z* 261 and 259, respectively), of CO (*m/z* 259 -28 = 231, and 261-28 = 233). The existence of these compounds is nutritionally important due to their helpful effect on inflammatory processes and the cardiovascular system (Kerwin et al., 1996).

### 3.2 Quantitative analysis of *S. asper* extracts

A quantitative investigation of *S. asper* extracts obtained from edible parts was performed on the main specialized metabolites, and the data from the quantitative analysis are listed in **Figure 1B**. This is the first report regarding the quantification of *S. asper* metabolites and was obtained by comparing the data with the reference standard calibration curves: rutin (concentration range 10–1000 ng/mL) to quantify quercetin derivatives, luteolin (concentration range 10–1000 ng/mL) to quantify flavonoid aglycones, luteolin 7-*O*- glucoside and apigenin 7-*O*-glucoside (concentration range 10–1500 ng/mL) to quantify luteolin and apigenin derivatives, aesculetin and roseoside (concentration range 10–1000 ng/mL), to quantify coumarins and glycoside, chlorogenic acid (concentration range 10–3500 ng/mL) to quantify phenolic acid derivatives, and linolenic acid (concentration range 10–4000 ng/mL) to quantify fatty acids and their hydroxylated derivatives. Results showed that apigenin glucuronide and luteolin glucuronide are the most representative flavonoids in all the extracts. Within the fatty acids, the abundant ones were trihydroxy-octadecadienoic acid and trihydroxy-octadecenoic acid, which showed the highest content in the raw U and cooked U extracts, together with the high concentration of the two hydroxy C18:2 fatty acid isomers. In general, the extracts obtained from cooked plants seem to be richer, especially in polyphenols. This could probably be due to cellular walls and compartments’ disruption during cooking with the consequent release of dietary fiber-bound polyphenols (Palermo et al., 2014). The quantitative profiles revealed that phenolic acid derivatives, primarily chlorogenic acid, chicoric acid, and caftaric acid, and unsaturated fatty acids were the most abundant compounds. Phenolic acids were mainly 3,4-dihydroxycinnamic acid (HCA) derivatives, which, together with the presence of unsaturated hydroxylated fatty acids, were known to have a great biological meaning. The essential amino acids were also quantified using the LC-MS/MS based method in order to discriminate between the different extraction methods (ultrasound and microwave extractions) and to compare the raw and cooked plant matrices. The results obtained (**Table 2**, **Figure 1B**) showed that all hydroalcoholic extracts are very rich in essential amino acids; in particular, isoleucine, leucine, and threonine are the most abundant. The presence of essential amino acids confers high nutritional value to *S. asper*.

### 3.3 Content of polyphenols and antioxidant properties of *S. asper* extracts

The cooked leaves of *S. asper* reported the highest polyphenols content (43.85 ± 0.43 mg GAE/g) when MW extraction was applied (**Table 3**). The content of specialized metabolites is related to the antioxidant activity since the cooked MW extract showed the highest radical-scavenging activity (21.72 ± 2.49 mgTE/g) and reducing power (29.63 ± 2.14 mgTE/g). Results of antioxidant activity and TPC were used to calculate Relative Antioxidant Capacity Index (RACI), and corroborating previous results, cooked MW showed the highest value (**Table 3**).

**Table 3:** Total phenolic content (TPC), and antioxidant activity of *S. asper* extracts and SAD, in solution or in eudragit-coated liposomes, and empty eudragit-coated liposomes.

	TPC *mg GAE/g* *(mg GAE/mL)*	DPPH *mg TE/g* *(mg TE/mL)*	FRAP *mg TE/g* *(mg TE/mL)*	RACI	α-amylase inhibition IC_50_, µg/mL
Raw U	20.08 ± 3.54^b^	15.67 ± 1.96^b^	9.70 ± 0.28^c^	-0.88	543.30 ± 5.59 ^c^
Cooked U	20.51 ± 1.18^b^	19.47 ± 1.56^b^	16.34 ± 2.05^b^	-0.11	312.30 ± 1.56 ^b^
Raw MW	20.21 ± 2.47^b^	17.88 ± 1.87^b^	14.94 ± 1.85^b^	-0.38	401.70 ± 19.73 ^b^
Cooked MW	43.85 ± 0.43^a^	21.72 ± 2.49^a^	29.63 ± 2.14^a^	1.37	127.80 ± 17.81^a^
SAD extract	63.36 ± 3.73 a (1.69 ± 0.10^a^)	209.56 ± 19.14 a (9.00 ± 0.94^a^)	1540.77± 110.28 ^b^ (12.33 ± 0.88^b^)		114.60 ± 1.08
SAD eu-liposomes	79.17 ± 6.03 ^a^ (2.02 ± 0.02^a^)	266.58 ± 23.53 a (11.46 ± 1.10^a^)	2526.34± 151.25 a (20.35 ± 2.85^a^)		nd
Empty eu-liposomes	(0.26 ± 0.04^b^)	(4.15 ± 1.06^b^)	(1.52 ± 0.22^c^)		nd
Acarbose					3.76 ± 0.20

mg GAE/g (mL), mg gallic acid equivalents per gram (mL) of dried extract; mg TE/g, mg of trolox equivalents per gram of dried extract; IC_50_, half maximal inhibitory concentration; RACI, Relative Antioxidant Capacity Index; Raw U, Ultrasound assisted extraction of raw leaves; Cooked U, Ultrasound assisted extraction of cooked leaves; Raw MW, Microwave assisted extraction of raw leaves; Cooked MW, Microwave assisted extraction of cooked leaves. Different letters (a–c) indicate significant differences among extracts (p ≤ 0.05; Tukey’s test).

nd, not determined.

### 3.4 Hypoglycemic activity of *S. asper* edible part extracts

All the extracts were tested to evaluate their hypoglycemic potential; data were expressed as IC_50_ (µg/mL) and compared with acarbose (**Table 3**). It is possible to observe that both extracts from cooked leaves showed an interesting *α*-amylase inhibitory activity. In particular, cooked MW reported the highest activity, as inferred by its IC_50_ of 127.80 ± 18.71 µg/mL. None of the extracts inhibited *α*-glucosidase enzyme at the tested concentrations.

### 3.5 Vesicle characterization

SAD eudragit-coated liposomes were produced, characterized, and compared with empty eudragit-coated liposomes, SAD uncoated liposomes and empty uncoated liposomes. **Table 1** reports the light scattering results. Empty uncoated liposomes displayed small size (80 nm), good homogeneity (P.I. 0.25), and positive charge (+8 mV) due to stearylamine. The loading of SAD did not affect these values significantly (*p* > 0.05). On the other hand, the eudragit coating induced an increase in size and inhomogeneity (ca. 100 nm and P.I. > 0.3; *p* < 0.01) and a decrease in zeta potential (+6 mV; *p* < 0.05) due to anionic eudragit. The entrapment efficiency of the eudragit-coated liposomes, calculated as a function of two abundant components of SAD (i.e., luteolin-glucuronide and apigenin-glucuronide), was very high (>90%; **Table 1**). The behaviour of the SAD eudragit-coated liposomes and SAD uncoated liposomes under pH and ionic strength conditions that simulate the gastrointestinal environment was studied (**Table 1**). SAD uncoated liposomes incubated at acidic pH displayed an increased size (~97 vs. 80 nm; **Table 1**) and polydispersity (P.I. ~0.3), while SAD eudragit-coated liposomes remained unchanged (~94 nm and P.I. ~0.3). SAD uncoated liposomes were found to be even more susceptible and prone to destabilization (i.e., aggregation) when incubated at neutral pH since the average size was above 100 nm. SAD eudragit-coated liposomes showed no remarkable variations instead. Fluctuations of zeta potential were detected as a function of the composition of the two media (i.e., protons or salts). Overall, the results demonstrate that the eudragit coating protected the vesicles and increased their physical stability.

### 3.6 Total phenolic content, antioxidant activity, and inhibition of α-amylase enzyme of SAD

The total phenolic content of SAD, both in solution and in eudragit-coated liposomes, was comparable (**Table 3**). This demonstrates that the nanoformulation process did not alter the extract’s phenolic content and antioxidant activity. The SAD extract showed antioxidant activity of 9.00 ± 0.94 mg TE/mL and 12.33 ± 0.88 mg TE/mL for scavenging activity and reducing power, respectively. The presence of phosphatidylcholine in the liposomal formulation conferred a slight antioxidant activity, in particular for the reducing power by FRAP assay (**Table 3**), but no significant differences were found for the scavenging activity. SAD solution inhibited the *α*-amylase enzyme with an IC_50_ value of 114.60 ± 1.08 µg/mL. The *α*-amylase and glucosidase inhibition could not be determined for the liposomal formulations due to the turbidity of reaction mixture and interferences with the spectrophotometric measurement.

### 3.7 STC-1 cell viability

**Figure 2:**
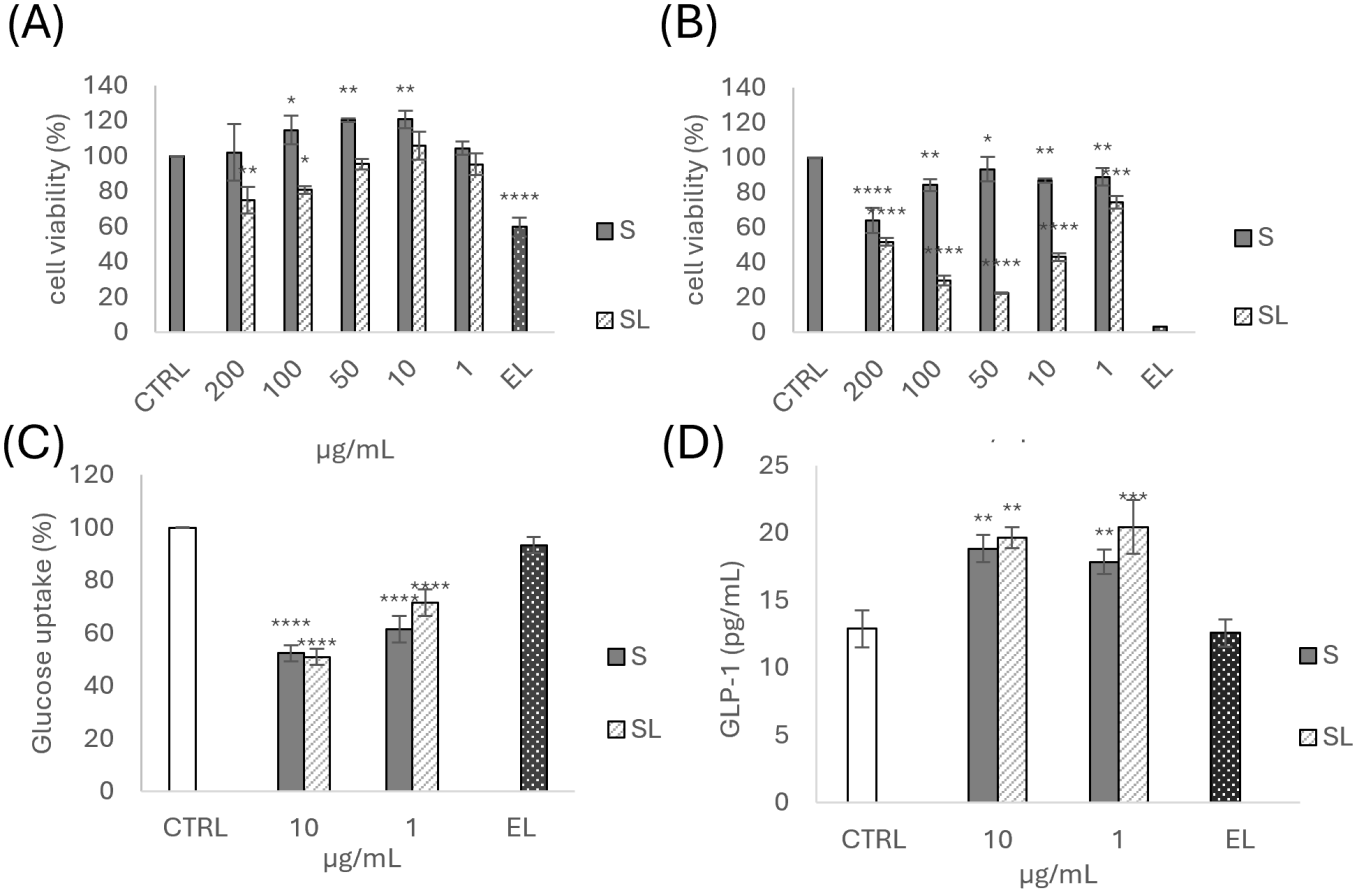
Cell viability was evaluated in STC-1 cells after **(A)** 2 h and **(B)** and 24 h of treatment with different concentrations of *S. asper* SAD extract and eudragit-coated liposomes; **(C)** Glucose uptake was measured on STC-1 cells treated for 2 h with different concentrations of *S. asper* SAD extract and eudragit-coated liposomes by using the 2-NBDG fluorescent glucose analog; **(D)** GLP-1 secretion was measured in STC-1 cells treated for 2 h with different concentrations of *S. asper* extract and eudragit-coated liposomes. Data are expressed as the mean ± SD of three independent experiments (n = 3) and were analyzed by one-way ANOVA followed by Tukey’s post-hoc test. **** p<0.0001, ***p<0.001, **p<0.01, * p<0.05 vs CTRL (100% viability). S, *S. asper* SAD extract solution; SL, *S. asper* SAD extract eudragit-coated liposomes; EL, empty eudragit-coated liposomes.

The SAD and its liposomal formulation effect on cell viability were evaluated in intestinal STC-1 cells using the colorimetric MTT assay. The treatment for 2 h with SAD extract at different concentrations (1-200 µg/mL) did not show marked changes in the active metabolism of the viable cells in converting MTT into formazan compared to the control, even at the highest tested concentration, as shown in **Figure 2**. The extract incorporation into eudragit-coated liposomes induced a slight reduction in cell viability, which became statistically significant at a concentration ≥ 100 µg/mL, though still being >70%. It has to be noted that such concentration is very high for a vesicular formulation applied in cell culture. In addition, in this specific case, the cytotoxicity can also be due to the stearylamine present in the formulation, as suggested by the low cell viability value detected for empty eudragit-coated liposomes (60% of cell viability, obtained by using the dilution for the 200µg/mL of SAD; **Figure 2A**). Stearylamine was found to be responsible for inducing apoptosis in a concentration- and time-dependent manner (Caddeo et al., 2019). Indeed, these effects were more evident after 24 h of treatment (**Figure 2B**). Nevertheless, 2 h is the ideal exposure time period for the following glucose-related experiments, and thanks to the viability assessment, the proper *S. asper* concentration range (1-10 µg/mL) was identified.

### 3.8 Intestinal glucose uptake and GLP-1 secretion from STC-1 cell line

The SAD effect, in solution or as liposomal formulation, on intestinal glucose uptake was evaluated by the 2-NBDG fluorescent probe in STC-1 cells. As shown in **Figure 2**, the treatment with the extract significantly (*p* < 0.001) reduced intestinal glucose absorption by about 50% at the highest tested concentration (10 µg/mL) with a significant inhibition (about 30% reduction), even at the lower concentration (1 µg/mL), compared to the untreated cells (CTRL). Furthermore, the liposomal formulation of the extract did not alter the effect of the extract, confirming its activity at all concentrations. The empty eudragit-coated liposomes (EL) showed no effect on glucose uptake compared to untreated control cells (CTRL).

The involvement of SAD and eudragit-coated liposomes on GLP-1 secretion was evaluated in STC-1 cells using a specific ELISA kit. Since the inhibition of intestinal glucose uptake was observed even at low concentrations, the cells were treated with 1 and 10 µg/mL of extract for 2 h, which were found to not affect cell viability. As reported in **Figure 2**, the extract increased GLP-1 secretion compared to untreated cells, and the effect was improved when the extract was delivered by eudragit-coated liposomes, especially at the lower concentration, nearly doubling the release (20.44 ± 1.98 pg/mL) compared to the control (12.89 ± 1.30 pg/mL). Empty eudragit-coated liposomes showed no statistically different GLP-1 secretion compared to untreated control cells (CTRL).

### 3.9 Economic valorisation of *S. asper* (L.) Hill

The potential valorisation action involves encouraging the harvesting of *S. asper* from abandoned land and promoting its marketing at local markets. This can be done by setting up cooperation schemes between agricultural consortia and owners of uncultivated land.

Also, with a view to possible cooperation schemes, adopting payment for ecosystem services (PES) mechanisms could incentivize the provision of conservation and protection services for WEPs According to Payment for Ecosystem Services (PES) mechanisms, external ecosystem service recipients engage in payments, direct, contractual and conditional, to local landowners and users. This payment system aims to encourage practices that guarantee the preservation and restoration of ecosystems. Considered a highly promising innovation in conservation, this scheme has been regarded as the most significant development since the establishment of the Convention on Biological Diversity in Rio in 1992. However, according to Narloch et al. (2011), the application of PES has not been adequately extended to agrobiodiversity preservation, specifically wild agrobiodiversity (Narloch et al., 2011). To close this gap, the authors proposed the concept of “payments for agrobiodiversity conservation services” (PACS) as a similar solution to PES for the landraces and other local crop varieties loss. According to this mechanism, governments, companies, or organizations can unilaterally finance PES programs for the conservation of wild species (Tyack et al., 2020), while the beneficiaries of such programs are farmers, private landowners or anyone who promotes the conservation of WEPs and, in the specific case of *S. asper*, harvesting, and marketing at local markets.

Since the cultivation of *S. asper* and other WEPs leads to a significant profit increase for agricultural operators, creating WEP gardens may be a possible means of valorisation. In addition to bringing economic benefits, it is an ecologically and socially sound strategy that favors both the conservation of plant biodiversity and the mitigation of health problems related to insufficient nutritional diversity. Furthermore, enhanced use of WEPs, which are traded locally and are more resistant to spoilage than other foods and crops, would also contribute to reduce food waste (Bacchetta et al., 2016).

To estimate the cost (a) of labour, an analysis was developed on the productivity of the individual worker who is able to harvest 3 seedlings per minute (and thus 5.4 kg of *S. asper* in one hour) on a field with the already indicated agricultural yield of 200 seedlings per 100 m^2^. Therefore, 111 hours of work are required per harvest, i.e. 4 workers each working for about 28 hours. Considering that the hourly wage for a fixed-term agricultural labourer is 8.72 €/hour for first-level labourers (source: Fund for the integration of various agricultural diseases and assistance – *Cassa per l’integrazione malattie ed assistenze agricole varie*, C.I.M.A.V., 2023), the annual costs (a) for labour amount to 2,907 €/ha. The cost (b) for transporting the produce to the sales market is estimated on the assumption that the products’ distribution can occur in local markets located, on average thirty, kilometres away from the harvesting grounds. Considering that one hectare of land produces 180 quintals of *S. asper* in a year and that a curtain-sided van has a capacity of 10 quintals, each harvest is distributed to six markets. Since the unit cost for transport to market is 0.80 €/km, as defined by the Italian road haulage company operating cost tables for category A vehicles (source: Ministry of Infrastructure and Transport, 2022), for every 10 quintals of *S. asper* the transport cost is 48 €. It follows that the annual costs for transport to the sales market, again referring to one hectare of land, amount to 864 €. In view of the financial items that contribute to the formation of the financial plan, compensation to the landowners by the consortium (c) equal to 5% of the revenues from the sale of the productions, thus amounting to 270 €/year·ha, is assumed to be fair.

Revenues are estimated according to the yield (Kg/ha· year) of *S. asper*. Based on direct surveys of farmers, an average yield of 200 seedlings per 100 m^2^ per harvest is estimated. Considering three harvests per year and depending on the average weight of 30 g per seedling, this results in an annual harvest of 1,800 kg per hectare. Since the reference market records an average selling price of *S. asper* of 3.00 €/kg, annual revenues amount to 5,400 €/ha. Data on production costs and revenues are summarised in **Table 4**, showing that the marketing of *S. asper* results in a profit for the operators of the consortium. This profit is worth 1.359 €/ha· year, which represents 33.6% of the total production costs. Therefore, the strategy for valorising and marketing *S. asper* based on a cooperation mechanism between the consortium members and the owner of uncultivated land is financially sustainable.

**Table 4:** Annual profits from the commercialisation of *S. asper*.

Harvest period	Spring
	Autumn
Labour costs (for harvesting)	8.72 €/hour for first level worker
Labour costs (for harvesting)	0.80 €/km
(a) Harvesting costs [€/year·ha]	2,906.67
(b) Cost of transport to the selling market [€/year·ha]	864.00
(c) Compensation for the landowner [€/year·ha]	270.00
**Total Costs [€/year·ha]**	**4,040.67**
Unit weight plant *S. asper* [g]	30
Productivity *S. asper*	
− N. plants/100 m^2^	200
− N. plants/ha**·**year	60,000
− Kg/ha**·**year	1,800
Sale price *S. asper* [€/kg]	3.00
**Revenues from sales [€/year·ha]**	**5,400**
**Profit [€/year·ha]**	**1,359**

The bold values refer to costs, revenues and profit.

## 4 Discussion

*S. asper* is mainly known as a foodstuff, but it is also a potential source of specialized metabolites with health benefits. However, like other WEPs, not only is it not sufficiently exploited, but its harvest is recently threatened by the increasing spread of abandoned land, which is particularly frequent in marginalised areas of Mediterranean regions due to increasing depopulation. In economic terms, it is shown that if appropriately exploited, the commercialisation of *S. asper* can: (i) contribute to improving the well-being of rural and urban communities and (ii) bring economic returns to farmers and landowners. In this regard, several strategies and actions can be taken for the economic valorisation of *S. asper* and, more generally, of WEPs. Scientific efforts are recognised as essential tools to preserve native wild species and the value of ancient culinary traditions.

In this study, the LC-HRMS analyses showed the presence of flavonoids, phenolic acids, and unsaturated fatty acids, besides C13-norisoprenoid glycosides and coumarins in traces, in *S. asper* extracts. Quantitative analyses of specialized metabolites displayed a higher content in extract obtained from cooked edible leaves, especially of flavonoids, phenolic acids and carotenoids, probably due to a disruption of cellular compartments occurring during cooking, which promotes the release and extractability of compounds (Fratianni et al., 2021). Previous studies confirmed that the brief boiling process increased polyphenols content, flavonoids, and antioxidant properties in green leafy vegetables such as *Spinacia oleracea* L, *Ipomoea aquatic* Forssk, *Basella rubra* L., and *Amaranthus gangeticus* L (Hossain et al., 2017). A prolonged boiling process (from 5 to 10 min) reduced the phytochemical composition and nutritional properties of *Urtica dioica* leaves (Sharma et al., 2022). These results could valorise the ancient culinary traditions and support the consumption of *S. asper* for its beneficial effects on health.

In recent times, there has been a growing emphasis on environmental pollution avoidance and the rationalization of the agro-industrial chain. This has led to increased interest in exploring the potential utilization of leftover vegetable materials within the framework of a circular economy. However, using vegetable waste directly as animal feed is challenging due to certain components in the waste that can cause animal intolerance. Additionally, the presence of bioactive substances, such as polyphenols, can hinder the use of composting methods due to their well-known properties of inhibiting germination. Several studies demonstrated that by-products generated during vegetable processing contain substantial quantities of proteins, lipids, and sugars, along with specialized metabolites, and, therefore, they could be an abundant and cheap market source to obtain high-added value products potentially useful as healthy products and functional foods. Moreover, the main specialized metabolites of *Cichorieae* tribe (Asteraceae family) have been demonstrated to possess several healthy properties (antioxidant, anti-inflammatory, hepatoprotective, and antidiabetic). *S. asper* extract from discarded leaves was found to be rich in bioactive compounds and possess stronger antioxidant activity than the edible part of the plant, therefore, in addition to the nutritional potential for the rediscovery of ancient dishes, developing a nanoformulation of *S. asper* extract from discarded leaves could contribute to an economic return. Eudragit-coated liposomes were characterized by a very high entrapment efficiency and an optimal resistance to the gastrointestinal environment’s harsh conditions (i.e., pH variations and high ionic strength), thanks to the gastro-resistant eudragit coating. The evaluation of the potential hypoglycemic effect of *S. asper* extract was carried out on intestinal STC-1 cell lines representing a model cell line for gut hormones secretion and glucose uptake studies due to their common features to l-enteroendocrine cells (McCarthy et al., 2015). The extract showed no cytotoxic effect against STC-1 after 2 and 24 h of treatment. Wang et al. also reported the absence of cytotoxic effect of the ethyl acetate aerial part fraction on RAW 264.7 cells (Wang et al., 2015). Empty eudragit-coated liposomes showed reduced cell viability after 24 h of treatment, likely due to the presence of stearylamine, which is used as positive charge-inducer that allows the electrostatic interaction with negatively charged eudragit. Nevertheless, the effect was mitigated when the *S. asper* extract was incorporated into the vesicles (Caddeo et al., 2019). Moreover, this effect was far less marked after 2 h of exposure, especially at concentrations ≤ 100 µg/mL, thus suggesting a safe use of the nanoformulation for hypoglycemic studies. The determination of the safety of the formulation represents a crucial step that offers the possibility of creating gastro-resistant models capable of bypassing the gastric tract and guaranteeing a site-specific release in the intestinal tract. In fact, it is well known that some natural compounds like polyphenols, strongly related to the hypoglycemic effects, possess low bioavailability and stability under the conditions of the digestive tract (Krook and Hagerman, 2012). Improving their stability with a gastro-resistant formulation, such as that offered by eudragit-coated liposomes, would enhance intestinal absorption. The current study evaluated the *S. asper* discarded leaves extract for its functions associated with hypoglycemic effect through two different assays. The inhibition of glucose uptake in the intestinal tract could represent a key strategy for diabetes treatment. It is mediated by the action of two transporters, sodium-dependent glucose cotransporters 1 (SGLT-1), located in the brush border membrane, and glucose transporter 2 (GLUT2), expressed on the basolateral membrane. An upregulation of these transporters is observed in type 2 diabetes mellitus (Ontawong et al., 2021). The search for new natural substances with an inhibitory action is necessary to fight this disease. The *S. asper* SAD extract exhibited a significant glucose uptake inhibition compared to untreated cells, in a dose-dependently, and interestingly, the activity was maintained in the liposomal formulation.

To verify *S. asper’s* hypoglycemic effect, its activity in inducing GLP-1 secretion was also evaluated. GLP-1 is an incretin hormone produced by enteroendocrine L cells found throughout the gastrointestinal mucosa. Its role in diabetes treatment is significant, as it induces insulin secretion, suppresses glucagon secretion, slows down gastric emptying, and decreases appetite and food intake (Grill, 2020). The secretion of GLP-1 has been shown to be markedly reduced in type 2 diabetes (Nauck et al., 2021). The obtained results indicate a marked increase in GLP-1 levels at the tested concentrations of *S. asper* extract, and its release was enhanced when the extract was nanoformulated. This demonstrates that eudragit-coated liposomes can be a new delivery formulation that protects the *S. asper* extract and potentiates its bioactivities, even at lower concentrations compared to the free extract.

However, some limitations at the current stage need to be addressed in a future work. First, the phytochemical profile of *S. asper* will need to be further investigated through GC-MS analysis for fatty acids estimation. Second, biological studies will have to be carried out on the compounds present in greater quantities to evaluate which of them is responsible for the activities found so far.

In conclusions, this study could contribute to the rediscovery of ancient dishes along with the preservation and sustainable use of biological diversity. Considering the potential positive impact of *S. asper* on human health and the environment, it is increasingly necessary to develop a participatory and holistic approach to the use of WEPs by involving scientists, farmers’ associations, and rural communities in the rediscovery of the nutritional and agro-ecological properties of wild herbs. In economic terms, the cultivation of *S. asper* could significantly increase profits for agriculture operators. Several efforts, both scientific and political, are still needed to foster the dissemination of WEPs, including *S. asper*, and make them recognised as essential elements of the human diet and economic benefits.

## Data availability statement

The data presented in the study are deposited in the Zenodo repository, https://doi.org/10.5281/zenodo.13142269.

## Ethics statement

Ethical approval was not required for the studies on humans in accordance with the local legislation and institutional requirements because only commercially available established cell lines were used. Ethical approval was not required for the studies on animals in accordance with the local legislation and institutional requirements because only commercially available established cell lines were used.

## Author contributions

VP: Data curation, Formal analysis, Investigation, Writing – original draft. VS: Data curation, Software, Validation, Writing – original draft. IF: Formal analysis, Investigation, Validation, Writing – original draft. NB: Formal analysis, Investigation, Writing – original draft. AV: Investigation, Methodology, Writing – review & editing. ND: Conceptualization, Funding acquisition, Writing – review & editing. LM: Funding acquisition, Resources, Validation, Writing – review & editing. AN: Conceptualization, Data curation, Writing – review & editing. GM: Formal analysis, Investigation, Writing – review & editing. AF: Funding acquisition, Data curation, Writing – review & editing. CC: Investigation, Validation, Writing – review & editing, Conceptualization.
